# Chemical Comparison of Two Drying Methods of Mountain Cultivated Ginseng by UPLC-QTOF-MS/MS and Multivariate Statistical Analysis

**DOI:** 10.3390/molecules22050717

**Published:** 2017-04-30

**Authors:** Xin-fang Xu, Shu-ya Xu, Ying Zhang, Hui Zhang, Meng-nan Liu, Huan Liu, Yan Gao, Xue Xue, Hui Xiong, Rui-chao Lin, Xiang-ri Li

**Affiliations:** 1 School of Chinese Materia Medica, Beijing University of Chinese Medicine, No. 6 Wangjing zhonghuannan Road, Beijing 100102, China; xuxinfang007@163.com (X.-f.X.); xushuya11@163.com (S.-y.X.); zhang0312ying@163.com (Y.Z.); zh19930503@sina.com (H.Z.); 20160931810@bucm.edu.cn (M.-n.L.); 20150931937@bucm.edu.cn (H.L.); 20150931752@bucm.edu.cn (Y.G.); sherry.xue@bucm.edu.cn (X.X.); xionghui@bucm.edu.cn (H.X.); linrch307@sina.com (R.-c.L.); 2 Beijing Key Laboratory for Quality Evaluation of Chinese Materia Medica, Beijing University of Chinese Medicine, No. 11 North Third Ring Road, Beijing 100029, China

**Keywords:** mountain cultivated ginseng (MCG), UPLC-QTOF-MS/MS, OPLS-DA, PCA, vacuum freeze-drying

## Abstract

In traditional Chinese medicine practice, drying method is an essential factor to influence the components of Chinese medicinal herbs. In this study, an ultra-performance liquid chromatography quadrupole time-of-flight tandem mass spectrometry (UPLC-QTOF-MS/MS)-based approach was used to compare the content of chemical compounds of mountain cultivated ginseng that had been natural air dried (LX-P) and vacuum freeze-dried (LX-L). Multivariate statistical analysis such as principal component analysis (PCA) and supervised orthogonal partial least squared discrimination analysis (OPLS-DA) were used to select the influential components of different samples. There were 41 ginsenosides unambiguously identified and tentatively assigned in both LX-L and LX-P. The results showed that the characteristic components in LX-P were ginsenoside Rb1, ginsenoside Rc, ginsenoside Rg6, dendrolasin, and ginsenoside Rb2. The characteristic components in LX-L were malonyl-ginsenoside Re, malonyl-ginsenoside Rb1, malonyl-ginsenoside Rc, malonyl-ginsenoside Rb1 isomer, malonyl-ginsenoside Rb2, malonyl-ginsenoside Rb3, malonyl-ginsenoside Rd isomer, gypenoside XVII, and notoginsenoside Fe. This is the first time that the differences between LX-L and LX-P have been observed systematically at the chemistry level. It was indicated that vacuum freeze-drying method can improve the content of malonyl-ginsensides in mountain cultivated ginseng.

## 1. Introduction

The root and rhizome of ginseng, *Panax ginseng* C.A. Meyer (Araliaceae), has been widely used as a traditional Chinese medicine and a functional food to prevent various diseases in the Orient [1]. Numerous research has shown that *Panax ginseng* possesses many pharmacological properties relating to the central nervous system [2], cardiovascular system [3], and aging process [4], which exhibits antioxidant [5], anticancer [6], and immunomodulatory effects [7]. The active components of ginseng are attributed to polysaccharides, ginsenosides, and volatile oil.

Mountain cultivated ginseng (MCG), which is grown in forests and mountains, can be considered to mimic mountain wild ginseng (MWG) [8]. Normally, MCG is harvested at the age of 10–20 years or more, and cultivated ginseng (CG) is often collected after 4–7 years [9]. Pharmacopoeia of the People’s Republic of China also classified ginseng into CG and MCG groups [10]. Nevertheless, as a substitute of MWG, MCG is of better quality than CG. Pharmacological researchers also have revealed that MCG has greater anticancer activities than CG [11]. More significantly, MCG can keep the balance of the ecological environment. Therefore, MCG has great potential value in clinical applications and environmental conservation.

The drying process is an essential factor for the quality of ginseng products, which directly relates to the variety of chemical components. After obtaining MCG samples, the drying process is necessary to reduce moisture content and water activity, which can keep it in a good quality for a long period of time. Besides, high moisture content of ginseng enhances microbiological growth, as well as enzymatic and non-enzymatic reactions that can result in a rapid deterioration of the ginseng and thus a reduction in its possible medicinal and commercial value [12]. The traditional drying process is drying ginseng in the natural air or in the sun. With the development of science and technology, many drying methods and equipment have been developed, such as forced air drying [13], vacuum freeze-drying [14], microwave drying [15], vacuum microwave drying [16], and far-infrared drying [17]. Natural air drying is a traditional drying method that was considered convenient and without cost. In recent years, vacuum freeze-drying, widely used in food and medicine fields, started to be used more widely for the preservation of Chinese herbs. Vacuum freeze-drying is a drying process in which the solvent contained inside the products is removed from a frozen solution by sublimation [18,19]. MCG, after vacuum freeze-drying, can keep consistent with its fresh condition in shape and color and contains more natural active components, which is often called active ginseng. Significant changes in the color, texture, and odor are directly related to the chemical content of ginseng samples. So, the chemical profiling of MCG that has been vacuum freeze-dried (LX-L) and natural air dried (LX-P) are important for the proper usage of ginseng.

In the past few decades, many analytical technologies have been frequently applied to identify and differentiate ginseng products. The studies for identifying ginseng in different drying methods focused on the chemical components including ginsenosides [20], polysaccharides [21], reducing sugars [22], amino acids [23] and volatile oil [24], etc. Among this research, the components of ginseng products were very similar in category and content. These methods were merely used to determine the major ginsenosides using high performance liquid chromatography (HPLC) and ultra-performance liquid chromatography tandem mass spectrometry (UPLC-MS), and to focus on cultivated ginseng (CG). There is no research to date that has systematically analyzed the difference between mountain cultivated ginseng (MCG) subjected to vacuum freeze-drying and natural air drying through identifying their chemical components.

In our study, we developed a sample profiling strategy combining UPLC-QTOF-MS/MS and multivariate statistical analysis (MVA) as the analytical tools to analyze the chemical contents of LX-P and LX-L. This strategy has the advantages of ultra-performance liquid chromatography (UPLC) for high resolution, high sensitivity, and high-speed separation, as well as time-of-flight mass spectrometry (TOF) for exact mass measurement capability. Moreover, MVA, especially the principle component analysis (PCA) and orthogonal projections from latent structures discriminant analysis (OPLS-DA), has been used to identify the differences between the samples. This method allows us to understand the subtle differences between LX-P and LX-L. More significantly, it can find the different marker components and their chemical structures to help identify mountain cultivated ginseng products easily. This is the first time that the differences between LX-P and LX-L have been systematically observed from the level of chemistry components.

## 2. Results and Discussions

### 2.1. UPLC-MS Analysis

**Figure 1:**
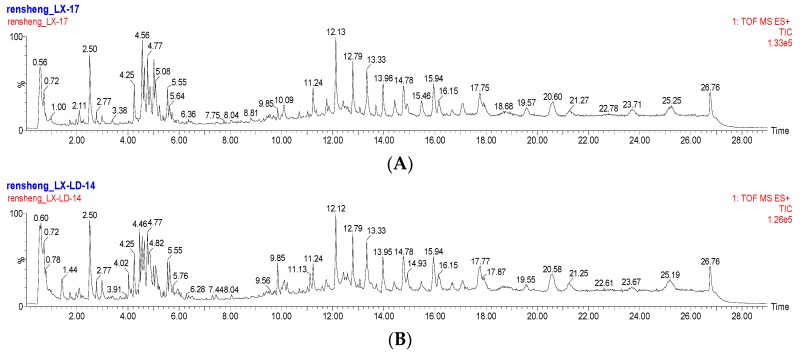
Representative based peak intensity (BPI) chromatograms of LX-P and LX-L samples. (**A**) Natural air dried ginseng (LX-P); (**B**) Vacuum freeze-dried ginseng (LX-L).

As shown in previous articles, the ACQUITY BEH C_18_ column has frequently been used to analyze ginsenosides from various ginseng products. Figure 1 shows the Based Peak Intensity (BPI) chromatograms obtained from the analysis of LX-L and LX-P in positive ion mode. The resultant peaks indicate that the components were complex in both MCG samples. There were 41 ginsenosides identified in LX-L and LX-P, including protopanaxatriol, panoxadiol, and their derivates. Among these ginsenosides, eight compounds were assigned by comparing them to standard ginsenosides, and 33 ginsenosides were identified by comparing their retention times and mass spectra with the reference compounds. The ginsenosides were further confirmed through ion fragmentation patterns. As illustrated in Table 1, ginsenosides were detected as protonated ions [M + H]^+^, sodium adduct ions [M + Na]^+^ and/or ammonium adduct ions [M + NH_4_]^+^ in the positive ion mode.

### 2.2. PCA Analysis

Due to the similar components contained in each sample, the differences between LX-L and LX-P were hard to identify only from the BPI chromatograms (shown in Figure 1). In this case, MVA was commonly applied to process the data, and we can clearly see the difference between LX-L and LX-P from the PCA score plot.

**Figure 2:**
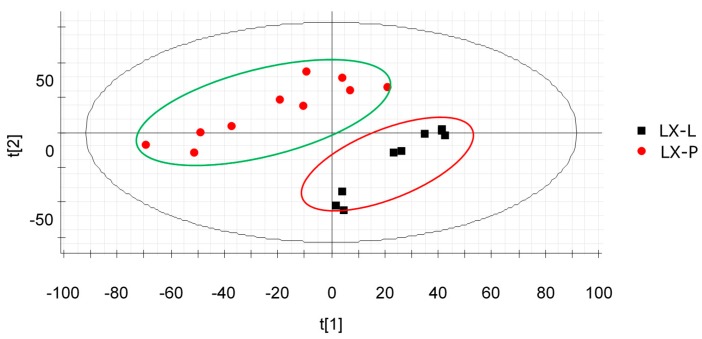
The principal component analysis (PCA) of LX-L and LX-P. LX-P: Natural air dried mountain cultivated ginseng; LX-L: Vacuum freeze-dried mountain cultivated ginseng.

**Figure 3:**
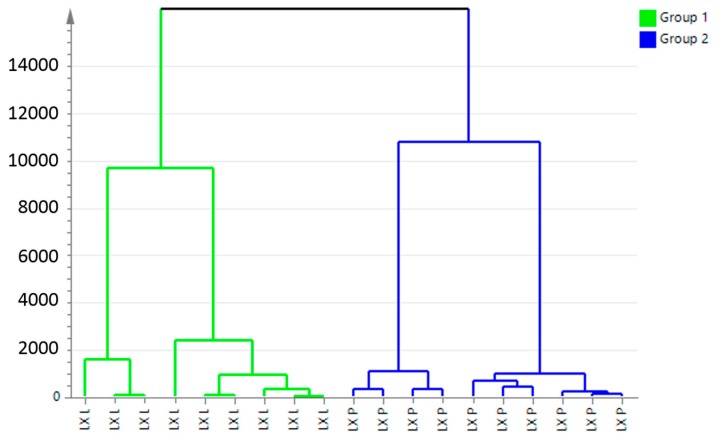
Hierarchical cluster analysis (HCA) dendrogram of LX-L and LX-P. LX-P: Natural air dried mountain cultivated ginseng; LX-L: Vacuum freeze-dried mountain cultivated ginseng.

A two-component PCA score plot of UPLC-QTOF-MS data was utilized to depict general variation of components among the mountain cultivated ginseng samples (Figure 2). The PCA scores plot in Figure 2 can be readily divided into two big clusters. The LX-L and LX-P samples were clearly separated by the principal component 1 (PC1). Figure 3 shows the hierarchical cluster analysis (HCA) dendrogram of mountain cultivated ginseng samples. It appears that the components of them are indeed differential.

### 2.3. Marker Ions Analysis

It is evident from Figure 2 that the samples were clearly clustered into two groups: one is LX-L, the other is LX-P, confirming that the components of LX-L and LX-P were indeed different in level and occurrence.

**Figure 4:**
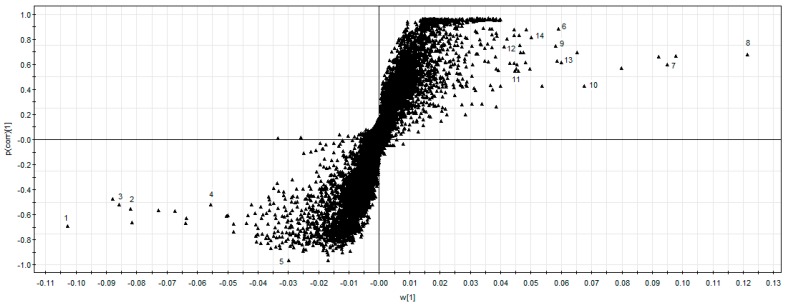
The S-Plot of LX-P and LX-L. 1 ion (t_R_ 4.56 min, *m*/*z* 1109.6176), 2 ion (t_R_ 4.76 min, *m*/*z* 1079.6058), 3 ion (t_R_ 4.98 min, *m*/*z* 1079.6069), 4 ion (t_R_ 5.56 min, *m*/*z* 947.5623) and 5 ion (t_R_ 10.00 min, *m*/*z* 219.1749); 6 ion (t_R_ 2.98 min, *m*/*z* 1033.5633), 7 ion (t_R_ 4.65 min, *m*/*z* 1195.6194), 8 ion (t_R_ 4.85 min, *m*/*z* 1165.6094) 9 ion (t_R_ 4.91 min, *m*/*z* 1195.6187), 10 ion (t_R_ 5.10 min, *m*/*z* 1165.6088), 11 ion (t_R_ 5.37 min, *m*/*z* 1165.6067), 12 ion (t_R_ 5.93 min, *m*/*z* 1033.5693), 13 ion (t_R_ 5.95 min, *m*/*z* 947.5623) and 14 ion (t_R_ 6.28 min, *m*/*z* 917.5517).

To explore the potential chemical markers that contributed most to the differences between LX-L and LX-P, UPLC-QTOF-MS/MS data were processed by supervised OPLS-DA. In the S-plot (Figure 4), each point of an exact mass retention time (EMRT) pair could be the potential markers. The X-axis and the Y-axis show the variable contributions and sample correlations, respectively. Therefore, the further away a data point is from the 0 value, the more it contributes to sample variance and the better its correlation from injection to injection. As shown in the S-plot in Figure 4, the first five ions, 1 ion (t_R_ 4.56 min, *m*/*z* 1109.6176), 2 ion (t_R_ 4.76 min, *m*/*z* 1079.6058), 3 ion (t_R_ 4.98 min, *m*/*z* 1079.6069), 4 ion (t_R_ 5.56 min, *m*/*z* 947.5623) and 5 ion (t_R_ 10.00 min, *m*/*z* 219.1749) at the lower left of the “S” were the ions from LX-P that contributed most to the differences between LX-L and LX-P. Analogously, the first nine ions, 6 ion (t_R_ 2.98 min, *m*/*z* 1033.5633), 7 ion (t_R_ 4.65 min, *m*/*z* 1195.6194), 8 ion (t_R_ 4.85 min, *m*/*z* 1165.6094) 9 ion (t_R_ 4.91 min, *m*/*z* 1195.6187), 10 ion (t_R_ 5.10 min, *m*/*z* 1165.6088), 11 ion (t_R_ 5.37 min, *m*/*z* 1165.6067), 12 ion (t_R_ 5.93 min, *m*/*z* 1033.5693), 13 ion (t_R_ 5.95 min, *m*/*z* 947.5623) and 14 ion (t_R_ 6.28 min, *m*/*z* 917.5517) in the top right corner of the “S” were ions from LX-L that contributed most to the difference between LX-L and LX-P. These ions could be used as potential chemical markers to distinguish LX-L from LX-P.

**Figure 5:**
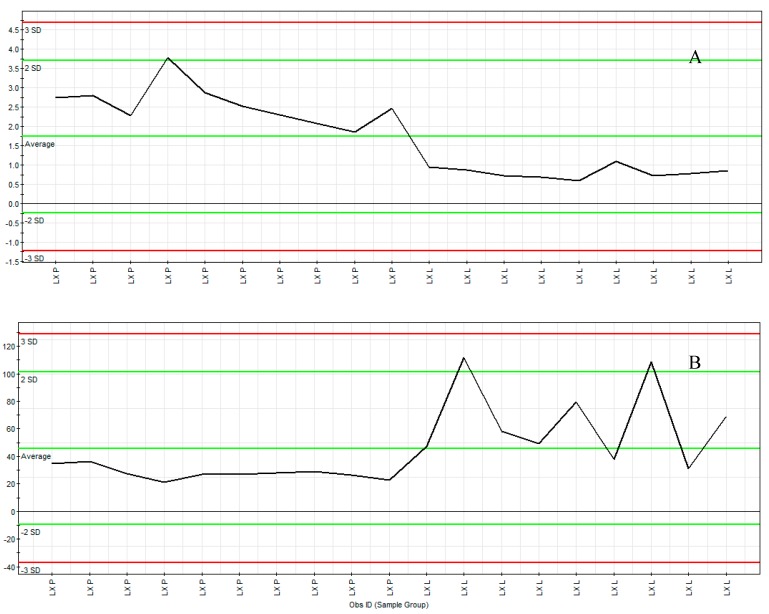
The ion intensity plot of LX-P and LX-L. LX-P: Natural air dried mountain cultivated ginseng; LX-L: Vacuum freeze-dried mountain cultivated ginseng; (**A**) Dendrolasin at *m*/*z* 219.1748 (t_R_ 10.00 min); (**B**) Mal-ginsenoside Rc at *m*/*z* 1165.6094 (t_R_ 4.85 min).

Moreover, we can further confirm these spectral variables using the ion intensity plot (Figure 5) which was generated by Marker Lynx software. It was the convenient instrument to aid the profiling of marker ions. The marker t_R_ 10.00 min, *m*/*z* 219.1748 (Figure 5A) was from the LX-P sample and the marker ion t_R_ 4.85 min, *m*/*z* 1165.6094 (Figure 5B) was from the LX-L sample. The representative ion intensity plot illustrated the abundance of marker ions t_R_ 10.00 min, *m*/*z* 219.1748 and t_R_ 4.85 min, *m*/*z* 1165.6094 over 19 MCG samples. The ions fulfilled the criteria of marker ions because they were found to have significant difference in the content levels of the samples.

### 2.4. Maker Ions Assignment

Once having obtained the potential markers, element composition calculation was performed for the target markers. The molecular formula of the markers can be easily obtained by calculating their accurate masses. The next step was to search against a database and use the retention times as correlation references to identify the markers. Finally, the structure of the markers was confirmed by the fragments which appeared in the high capillary electrophoresis (CE) scan. The results are in Table 2.

By matching the retention time and accurate mass with the published known compounds, ion 1 (t_R_ 4.56 min, *m*/*z* 1109.6176), ion 2 (t_R_ 4.76 min, *m*/*z* 1079.6058), 3 (t_R_ 4.98 min, *m*/*z* 1079.6069), ion 4 (t_R_ 5.56 min, *m*/*z* 947.5623), and ion 5 (t_R_ 10.00 min, *m*/*z* 219.1749) in the LX-P samples were identified as ginsenoside Rb1, ginsenoside Rc, ginsenoside Rb2, ginsenoside Rg6, and dendrolasin, respectively. Similarly, ion 6 (t_R_ 2.98 min, *m*/*z* 1033.5633), ion 7 (t_R_ 4.65 min, *m*/*z* 1195.6194), ion 8 (t_R_ 4.85 min, *m*/*z* 1165.6094), ion 9 (t_R_ 4.91 min, *m*/*z* 1195.6187), ion 10 (t_R_ 5.10 min, *m*/*z* 1165.6088), ion 11 (t_R_ 5.37 min, *m*/*z* 1165.6067), ion 12 (t_R_ 5.93 min, *m*/*z* 1033.5693), ion 13 (t_R_ 5.95 min, *m*/*z* 947.5623), and ion 14 (t_R_ 6.28 min, *m*/*z* 917.5517) in the LX-L samples were affirmed to be malonyl-ginsenoside Re, malonyl-ginsenoside Rb1, malonyl-ginsenoside Rc, malonyl-ginsenoside Rb1 isomer, malonyl-ginsenoside Rb2, malonyl-ginsenoside Rb3, malonyl-ginsenoside Rd isomer, gypenoside XVII, and notoginsenoside Fe, respectively.

After assigning the maker ions, we could easily find that mountain cultivated ginseng processed in different drying methods have a significant different in their chemical components. Malonyl-ginsenosides, which are naturally present in ginseng, were abundant in LX-L. However, LX-P contained a large number of major ginsenosides which were derived from malonyl-ginsenosides by natural air drying. This study indicated that the drying method is an essential factor to controlling the quality of mountain cultivated ginseng, and the vacuum freeze-drying method was found to improve the content of malonyl-ginsensides in mountain cultivated ginseng.

## 3. Materials and Methods

### 3.1. Ginseng Samples and Sample Processing

There were 19 MCG samples which were cultivated for 15 years before being collected from Ji’an city of the Jilin province of China. All these samples were fresh ginseng, which were then processed by natural air drying or by vacuum freeze-drying, respectively. All of these processed samples were identified by Professor Xiangri Li (School of Chinese Materia Medica, Beijing University of Chinese Medicine) and deposited in the specimen cabinet of traditional Chinese medicine of Beijing University of Chinese Medicine.

### 3.2. Sample Preparation

The dried roots were powdered to a homogeneous size, and sieved through a No. 65 mesh. The amount of 0.4 g of ginseng powder was accurately weighed and then placed in a triangular flask with 50 mL methanol, filled with a plug, weighed, and ultrasonic-extracted for 30 min. After cooling to room temperature, the loss of weight was replenished with methanol and then the sample was filtrated. Precision draw subsequent filtrate 25 mL and concentrated it into residue, which was then dissolved in methanol in a 10-mL volumetric flask. Finally, the extraction solution was injected into the UPLC system after being filtered through a 0.22-μm filter membrane.

### 3.3. Reagents

Fisher Optima grade acetonitrile, methanol, and isopropanol were purchased from Thermo Fisher Co. (Waltham, MA, USA). Formic acid and leucine enkephaline were purchased from Sigma Aldrich (St. Louis, MI, USA). Ultra-pure water was obtained in our laboratory via a Milli-Q water purification system (Millipore Corporation, Bedford, MA, USA). Ginsenoside Rg1, Re, Rb1, Rf, Rb2 and Rb3 standards were purchased from the National Institute for the Pharmaceutical and Biological Products (Beijing, China). Ginsenoside Rc, Rg2 standards were obtained from the Beijing Xiantong era Pharmaceutical Co. Ltd. (Beijing, China). The standards were dissolved in methanol and stored at 4 °C until analysis.

### 3.4. UPLC-Q-TOF Conditions

#### 3.4.1. Liquid Chromatography Conditions

UPLC separation was performed by an ACQUITY UPLC system (Waters Corporation, Milford, Massachusetts) with an ACQUITY UPLC BEH C_18_ column (100 mm × 2.1 mm, 1.7 μm). The column temperature was controlled at 40 °C. The flow rate was kept at 400 μL/min. The binary gradient elution solvent consisted of water with 0.1% formic acid (A) and acetonitrile (B). The UPLC elution conditions were optimized as follows: initially, A:B = 81:19; 0–7 min, A:B = 50:50; 7–12 min, A:B = 4:96; 12–13 min, A:B = 2:98; 13–25 min, A:B = 2:98; 25–26 min, A:B = 81:19; 26–29 min, A:B = 81:19. The total run time was 29 min, and the sample injection volume was 2 μL.

#### 3.4.2. Mass Spectrometry Conditions

MS detection was performed on a quadrupole orthogonal acceleration time-of-flight tandem mass spectrometer (Waters Synapt MS System). The data acquisition mode was MS^E^ and the ion polarity was set to positive mode (ESI^+^). The optimized condition was desolvation gas at 480.0 L/h at a temperature of 350 °C, cone gas at 50 L/h and source temperature at 120 °C, capillary and cone voltage at 3.0 kV and 20 V, respectively. The lock mass compound used was leucine enkephaline. The low-energy scan collision energy was set at 5 eV in order to collect information on the intact precursor ions, and high-energy scan energy was set at 20 eV–30 eV to obtain the fragment ions. The UPLC-MS data acquisition was controlled by Mass Lynx 4.1 Mass Spectrometry Software (Waters Corporation).

### 3.5. Data Processing Procedure

For post-acquisition data processing, the MVA such as PCA and OPLS-DA were performed by Marker Lynx XS, which is an application manager for Mass Lynx software. The structural elucidation was performed by the Mass Fragment tool provided by Mass Lynx.

#### 3.5.1. The PCA Scores Plot of Samples

From the chromatographic trace, we actually acquired three-dimensional (3-D) data which represented retention time, *m*/*z*, and intensity. It was necessary to convert each data point into a 2-D matrix, i.e., an exact mass retention time (EMRT) pair. After the EMRT 2-D matrix was obtained, the MVA interface was launched with all EMRT information automatically imported so that the extended statistics module PCA could be executed.

#### 3.5.2. The Scatter Plot (S-Plot) from OPLS-DA Analysis

The loading plot (S-plot) of every group pair was processed by OPLS-DA analysis. In the S-plot, the leading contributing EMRT pairs could be captured selectively so that a list of top contributing markers from each sample group was generated and saved as a text file.

#### 3.5.3. The Elemental Composition Calculation for the Targeting Markers

The matched elemental composition of markers was obtained by calculating the exact mass. Then, we searched against an existing database to acquire the chemical structure. Once the identity of a marker was tentatively identified, its fragment ions could be easily obtained by going back to the raw data file to investigate the high capillary electrophoresis (CE) scan of the samples. The fragment ions which we obtained through the Mass Fragment tool of Mass Lynx was used for elucidating the structure.

## 4. Conclusions

The multivariate statistical analysis (MAV) and UPLC-QTOF-MS/MS were combined to analyze mountain cultivated ginseng subjected to either natural air drying or vacuum freeze-drying. The combination of the high-resolution UPLC separation and high-resolution MS detection along with the multivariate statistical analysis (MAV) details of the samples proved able to identify and select the important marker ions in both samples, even at low concentration levels. As a result, this is the first time that the differences between LX-P and LX-L have been observed systematically at the level of their chemical components.

## Figures and Tables

**Table 1:** Characterization of ginsenosides in LX-L and LX-L using UPLC-QTOF-MS/MS.

No.	t_R_ (min)	Precursor Ion and/or Adduct Ions	Exact Mass [M + H]^+^	Error (ppm)	Formula	Identification
1	1.99	933.5476 [M + H]^+^	933.5423	5.6	C_47_H_80_O_18_	ginsenoside Re4
2	2.08	963.5582 [M + H]^+^, 980.5865 [M + NH_4_]^+^	963.5529	5.5	C_48_H_82_O_19_	notoginsenoside R3 isomer
3	2.11	933.5474 [M + H]^+^	933.5423	5.4	C_47_H_80_O_18_	notoginsenoside R1
4	2.50	947.5628 [M + H]^+^	947.5579	5.1	C_48_H_82_O_18_	ginsenoside Re
5	2.50	801.5038 [M + H]^+^	801.5000	4.7	C_42_H_72_O_14_	ginsenoside Rg1
6	2.77	887.5040 [M + H]^+^, 904.5305 [M + NH_4_]^+^	887.5004	4.0	C_45_H_74_O_17_	malonyl-ginsenoside Rg1
7	2.98	1033.5633 [M + H]^+^	1033.5583	4.8	C_51_H_84_O_21_	malonyl-ginsenoside Re
8	3.03	1033.5630 [M + H]^+^	1033.5583	4.5	C_51_H_84_O_21_	malonyl-ginsenoside Re isomer
9	4.02	1241.6609 [M + H]^+^, 1258.6971 [M + NH_4_]^+^	1241.6530	6.3	C_59_H_100_O_27_	ginsenoside Ra3/notoginsenoside R4/notoginsenoside Fa
10	4.13	1327.6656 [M + H]^+^, 1327.6980 [M + NH_4_]^+^	1327.6534	9.1	C_62_H_102_O_30_	malonyl-ginsenoside Ra3
11	4.25	801.5033 [M + H]^+^	801.5000	4.1	C_42_H_72_O_14_	ginsenoside Rf
12	4.37	1327.6666 [M + H]^+^	1327.6534	9.9	C_62_H_102_O_30_	malonyl-notoginsenoside R4
13	4.45	1211.6492 [M + H]^+^, 1228.6871 [M + NH_4_]^+^	1211.6425	5.5	C_58_H_98_O_26_	ginsenoside Ra2
14	4.56	1109.6176 [M + H]^+^, 1126.6500 [M + NH_4_]^+^	1109.6108	6.1	C_54_H_92_O_23_	ginsenoside Rb1
15	4.60	1327.6655[M + H]^+^	1327.6534	9.1	C_62_H_102_O_30_	malonyl-notoginsenoside Fa
16	4.65	1195.6194 [M + H]^+^	1195.6112	6.8	C_57_H_94_O_26_	malonyl-ginsenoside Rb1
17	4.77	1079.6058 [M + H]^+^	1079.6002	5.1	C_53_H_90_O_22_	ginsenoside Rc
18	4.77	1211.6507 [M + H]^+^, 1228.6721 [M + NH_4_]^+^	1211.6425	6.7	C_58_H_98_O_26_	ginsenoside Ra1
19	4.85	1165.6094 [M + H]^+^	1165.6006	7.2	C_56_H_92_O_25_	malonyl-ginsenoside Rc
20	4.85	1297.6490 [M + H]^+^	1297.6429	4.7	C_61_H_100_O_29_	malonyl-ginsenoside Ra2/Ra1
21	4.91	1195.6187 [M + H]^+^, 1212.9451 [M + NH_4_]^+^	1195.6112	6.2	C_57_H_94_O_26_	malonyl-ginsenoside Rb1 isomer
22	4.99	1079.6069 [M + H]^+^, 1096.6310 [M + NH_4_]^+^	1079.6002	6.2	C_53_H_90_O_22_	ginsenoside Rb2
23	5.10	1165.6088 [M + H]^+^, 1182.641 [M + NH_4_]^+^	1165.6006	7.0	C_56_H_92_O_25_	malonyl-ginsenoside Rb2
24	5.23	1151.6284 [M + H]^+^, 1168.6471 [M + NH_4_]^+^	1151.6213	6.1	C_56_H_94_O_24_	quinquenoside R1
25	5.24	1079.6069 [M + H]^+^, 1096.6312 [M + NH_4_]^+^	1079.6002	6.2	C_53_H_90_O_22_	ginsenoside Rb3
26	5.37	1165.6067 [M + H]^+^, 1182.641 [M + NH_4_]^+^	1165.6006	5.2	C_56_H_92_O_25_	malonyl-ginsenoside Rb3
27	5.41	1165.6085 [M + H]^+^, 1182.641 [M + NH_4_]^+^	1165.6006	6.7	C_56_H_92_O_25_	malonyl-ginsenoside Rb3 isomer
28	5.55	947.5621 [M + H]^+^, 964.5913 [M + NH_4_]^+^	947.5579	4.4	C_48_H_82_O_18_	ginsenoside Rd
29	5.56	767.4960 [M + H]^+^	767.4960	1.8	C_42_H_70_O_12_	ginsenoside Rg6
30	5.64	1033.5644 [M + H]^+^, 1050.590 [M + NH_4_]+	1033.5583	5.9	C_51_H_84_O_21_	malonyl-ginsenoside Rd
31	5.76	1121.6008 [M + H]^+^	1121.6108	−8.2	C_55_H_92_O_23_	ginsenoside Rs1
32	5.92	1033.5653 [M + H]^+^, 1050.590 [M + NH_4_]^+^	1033.5583	6.7	C_51_H_84_O_21_	malonyl-ginsenoside Rd isomer
33	5.95	947.5623 [M + H]^+^	947.5579	4.6	C_48_H_82_O_18_	gypenoside XVII
34	6.01	1121.6180 [M + H]^+^	1121.6108	5.9	C_55_H_92_O_23_	ginsenoside Rs2
35	6.12	1147.6347 [M + H]^+^	1147.6264	7.2	C_57_H_94_O_23_	ginsenoside Ra7
36	6.28	917.5440 [M + H]^+^	917.5474	−3.7	C_47_H_80_O_17_	notoginsenoside Fe
37	6.36	1147.6348 [M + H]^+^	1147.6264	7.3	C_57_H_94_O_23_	ginsenoside Ra8
38	6.40	767.4987 [M + H]^+^	767.4946	5.3	C_42_H_70_O_12_	ginsenoside F4
39	6.51	917.5518 [M + H]^+^	917.5474	4.7	C_47_H_80_O_17_	vinaginsenoside R16
40	7.29	785.5082 [M + H]^+^	785.5051	3.9	C_42_H_72_O_13_	ginsenoside Rg3
41	16.68	663.4530 [M + H]^+^, 685.4382 [M + Na]^+^	663.4472	8.7	C_38_H_62_O_9_	ginsenosde Rs6/Rs7

**Table 2:** Identified maker ions of mountain cultivated ginseng (MCG) in different drying methods.

No.	Identification	t_R_ (min)	Molecular Formula	Ion	Mean Measured Mass	Theoretical Exact Mass	Mass Accuracy (ppm)	Fragment Ions	Classification
1	ginsenoside Rb1	4.56	C_54_H_92_O_23_	[M + H]^+^	1109.6176	1109.6180	−0.4	929, 767, 605, 425	LX-P
2	ginsenosede Rc	4.76	C_53_H_90_O_22_	[M + H]^+^	1079.6058	1079.6002	5.1	929, 767, 605	LX-P
3	ginsenoside Rb2	4.98	C_53_H_90_O_22_	[M + H]^+^	1079.6069	1079.6002	6.2	929, 767, 605, 425	LX-P
4	ginsenoside Rg6	5.56	C_56_H_94_O_24_	[M + H]^+^	767.4960	767.4946	1.8	621, 459	LX-P
5	dendrolasin	10.00	C_15_H_22_O	[M + H]^+^	219.1748	219.1749	−0.5	203, 149	LX-P
6	mal-ginsenoside Re	2.98	C_51_H_84_O_21_	[M + H]^+^	1033.5633	1033.5583	4.8	1015, 853, 767, 605	LX-L
7	mal-ginsenoside Rb1	4.65	C_57_H_94_O_26_	[M + H]^+^	1195.6194	1195.6112	6.8	1109, 1015, 853, 835, 785, 605, 425	LX-L
8	mal-ginsenoside Rc	4.85	C_56_H_92_O_25_	[M + H]^+^	1165.6094	1165.6006	7.5	1187, 1079, 1015, 853, 835,605, 425, 411	LX-L
9	mal-ginsenoside Rb1 isomer	4.91	C_57_H_94_O_26_	[M + H]^+^	1195.6187	1195.6112	6.2	1109, 1015, 853, 785	LX-L
10	mal-ginsenoside Rb2	5.10	C_56_H_92_O_25_	[M + H]^+^	1165.6088	1165.6006	7.0	1079, 871, 853, 411	LX-L
11	mal-ginsenoside Rb3	5.37	C_56_H_92_O_25_	[M + H]^+^	1165.6067	1165.6006	5.2	1079, 871, 853, 411	LX-L
12	mal-ginsenoside Rd iosmer	5.93	C_51_H_84_O_21_	[M + H]^+^	1033.5653	1033.5583	6.7	947, 871, 785, 605	LX-L
13	gypenoside XVII	5.95	C_48_H_82_O_18_	[M + H]^+^	947.5623	947.5579	4.6	785, 767, 605, 443	LX-L
14	notoginsenoside Fe	6.28	C_47_H_80_O_17_	[M + H]^+^	917.5517	917.5474	−3.7	899, 785, 737, 605	LX-L

mal: malonyl; LX-P: Natural air dried mountain cultivated ginseng; LX-L: Vacuum freeze-dried mountain cultivated ginseng.
